# A Perspective on Developing a Plant ‘Holobiont’ for Future Saline Agriculture

**DOI:** 10.3389/fmicb.2022.763014

**Published:** 2022-05-06

**Authors:** Cheng-Gang Ren, Cun-Cui Kong, Zheng-Yi Liu, Zhi-Hai Zhong, Jian-Chao Yang, Xiao-Li Wang, Song Qin

**Affiliations:** ^1^Key Laboratory of Biology and Utilization of Biological Resources of Coastal Zone, Yantai Institute of Coastal Zone Research, Chinese Academy of Sciences, Yantai, China; ^2^Center for Ocean Mag-Science, Chinese Academy of Sciences, Qingdao, China; ^3^Yantai Academy of Agricultural Sciences, Yantai, China; ^4^College of Horticulture, Qingdao Agricultural University, Qingdao, China

**Keywords:** symbiosis, sustainable agriculture, saline soil, plant ‘holobiont’, common symbiotic pathway

## Abstract

Soil salinity adversely affects plant growth and has become a major limiting factor for agricultural development worldwide. There is a continuing demand for sustainable technology innovation in saline agriculture. Among various bio-techniques being used to reduce the salinity hazard, symbiotic microorganisms such as rhizobia and arbuscular mycorrhizal (AM) fungi have proved to be efficient. These symbiotic associations each deploy an array of well-tuned mechanisms to provide salinity tolerance for the plant. In this review, we first comprehensively cover major research advances in symbiont-induced salinity tolerance in plants. Second, we describe the common signaling process used by legumes to control symbiosis establishment with rhizobia and AM fungi. Multi-omics technologies have enabled us to identify and characterize more genes involved in symbiosis, and eventually, map out the key signaling pathways. These developments have laid the foundation for technological innovations that use symbiotic microorganisms to improve crop salt tolerance on a larger scale. Thus, with the aim of better utilizing symbiotic microorganisms in saline agriculture, we propose the possibility of developing non-legume ‘holobionts’ by taking advantage of newly developed genome editing technology. This will open a new avenue for capitalizing on symbiotic microorganisms to enhance plant saline tolerance for increased sustainability and yields in saline agriculture.

## Introduction

**FIGURE 1:**
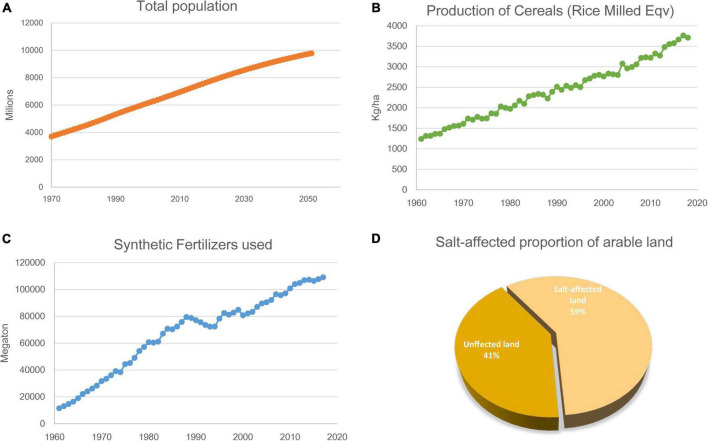
Global population, crop yield, synthetic fertilizers used and salt-affected proportion of arable land. **(A)** World population from 1970 to 2060. **(B)** Production of Cereals (Rice Milled Eqv) of the world since 1961 to 2018. **(C)** Consumption of synthetic nitrogen fertilizers of the world since 1961 to 2018. **(D)** Salt-affected proportion of arable land. All data taken from FAO-STAT (http://www.fao.org/statistics/en/). Figures ellaborated by the authors from FAOSTAT data.

The current world population of 7.8 billion is expected to reach 9.8 billion in 2050, increase by 25% in the next 30 years (Figure 1A). Global food production will need to increase as well. Historically, the highest global population growth rates, with increases of over 1.8% per year, occurred between 1955 and 1975, peaking to 2.1% between 1965 and 1970 (UN, 2019). In roughly coincident time frames, scientific and technical advances induced a series of innovations in farming that increased crop yields dramatically and were later known as the “Green Revolution” (GR, the 1950s to 1970s) (Wu et al., 2020). Part of the core operation of the GR was carrying out large-scale monoculture and using chemical pesticides, herbicides, and fertilizers (Liu et al., 2014; Nicolopoulou-Stamati et al., 2016). This farming method successfully increased the grain yield of the main crops (Figure 1B). However, its adverse consequences are becoming increasingly apparent. The inevitable consequence of large-scale monoculture is deep-rooted perennial species are replaced by shallow-rooted, annual species. It will increase leakage and groundwater recharge, leading to dissolved salts move toward the soil surface. Eventually, the soil becomes salinized (FAO, 2015). Going hand-in-hand, the increasing use of synthetic fertilizers poses hidden dangers to sustainable agricultural development and food security worldwide (Figure 1C). Therefore, achieving food security for the growing population amidst the gradual salinization of farmland is one of the most important missions for modern agriculture.

It is not economically or environmentally feasible to expand traditional agricultural practices to meet future demand. Therefore, there is an urgent need for alternative technologies to sustainably meet global food security requirements. One way to increase sustainable crop yields is to amplify the role of plant–microbe symbiosis. Symbiotic microorganisms such as arbuscular mycorrhizal (AM) fungi and rhizobia can significantly improve crop growth and vigor, nutrient utilization efficiency, and biological/abiotic stress resistance. If these effects could be used in saline agriculture, they could increase agricultural productivity and food quality sustainably, thereby bringing positive environmental, social, and economic results.

## Soil Salinization Affects Agriculture Globally

Since the beginning of industrialization, human activities have severely damaged the natural hydrological balance in many regions of the world. These activities affect the natural distribution of salt in various surface landscapes and ultimately lead to the deterioration of the natural and agricultural environment. On a global scale, soil salinization has become a growing threat to food production amidst increasing climate change. Soil salinization is commonly caused by climate changes (primary) or anthropogenic activity (secondary). Primary processes include weathering of mother rock, seawater deposition, and atmospheric deposition. Secondary processes include inadequate drainage, brackish water irrigation, and long-term continuous agricultural irrigation (Rengasamy, 2010). The area of primary salinization is estimated to be slightly under 1 billion ha. Secondary salinization has occurred on around 77 million ha, of which 58% is in irrigated areas; as much as 20% of all irrigated areas are estimated to be salt-affected within India, Pakistan, China, Iraq, and Iran (FAO, 2015; Abbaspour and Ashraf Vaghefi, 2019). About 5.2 billion ha of the world’s agricultural land is already salt-affected and not suitable for conventional crop farming (Figure 1D; Ahmed et al., 2016).

Soil salinization is caused by the excessive accumulation of ions in the soil, including calcium, magnesium, sodium, sulfate, and chloride ions, resulting in plant growth inhibition. Excessive salt interferes with the absorption of nutrients and water by plants, thereby disrupting the physiological processes necessary for plant growth and development (Munns, 2002). Therefore, salinization is an important factor causing land degradation and a major threat to non-renewable soil resources. Unfortunately, the salinization of farmland is continuing and is estimated to be expanding by 0.3–1.5 million hectares every year, resulting in crop yield losses in these areas of more than 20% (Porcel et al., 2011). Worldwide soil salinization will have a double impact on social and economic progress. With the continuous salinization of arable land, agricultural income and the world food supply will eventually suffer. It is estimated that 12–27.3 billion US dollars are lost annually due to reductions in crop productivity (Qadir et al., 2014).

## Symbiotic Microbes Can Help Plants Tolerate Salt Stress

Soil salinity affects the germination and growth of plants, and excessive salinity can cause severe yield reductions (Evelin et al., 2009). Excessive salinity has three negative effects on plants. First, the toxic effects of specific ions such as sodium and chlorine inhibit protein synthesis and damaged organelles, enzyme structures, and the system on which photosynthesis and respiration depend. Second, excessive salt can hinder nutrient absorption and/or transportation to shoots, resulting in nutrient deficiency in plants (Marschner, 2002; Evelin et al., 2009). Finally, too much salt in the soil reduces its osmotic potential and hinders water absorption by the root system, leading to physiological drought in the plant. In this state, the plant must reduce its internal osmotic potential to prevent water from entering the soil from the roots. Due to their immobility, when facing constant environmental stress plants not only develop their adaptive mechanisms, but also co-evolve with soil microorganisms to develop complex mechanisms to resist stress. For example, the interaction with symbiotic soil microorganisms such as rhizobium and AM fungi have a great impact on the salt stress tolerance of plants (Table 1; Marschner, 2002; Porcel et al., 2011; Ngom et al., 2016).

**TABLE 1:** Strains of AM fungi and rhizobia enhancing plant salinity tolerance.

Microorganisms inoculum	Plant species	References
**Mycorrhizal fungi**		
*Rhizophagus intraradices* [syn. *Glomus intraradices*] and *Funneliformis mosseae* [syn. *Glomus mosseae*]	Giant Reed (*Arundo donax* L.)	Romero-Munar et al., 2019
*Claroideoglomus etunicatum* [syn. *Glomus etunicatum*], *Rhizophagus intraradices* and *Funneliformis mosseae*	Cucumber (*Cucumis sativus* L.)	Hashem et al., 2018
*Funneliformis mosseae*	Hangbaiju (*Chrysanthemum morifolium*)	Wang et al., 2018
*Funneliformis mosseae*	*Sesbania cannabina*	Kong et al., 2017; Ren et al., 2018
*Rhizophagus irregularis*	Durum wheat (*Triticum durum* Desf.)	Fileccia et al., 2017
*Rhizophagus irregularis*	Black locust (*Robinia pseudoacacia* L.)	Chen et al., 2017
*Septoglomus deserticola* [syn. *Glomus deserticola*]	Sweet basil (*Osmium basilicum*)	Elhindi et al., 2017
*Rhizophagus irregularis*	Tomato (*Solanum lycopersicum*, cv. TT-115)	Khalloufi et al., 2017
*Funneliformis mossseae* and *Rhizophagus irregularis*	Pigeonpea (*Cajanus cajan*)	Pandey and Garg, 2017
*Claroideoglomus etunicatum*	Rice (*Oryza sativa* L.)	Porcel et al., 2016
*Funneliformis mossseae*	Pistachio (*Pistacia vera*)	Shamshiri and Fattahi, 2016
*Rhizophagus irregularis*	Cathay Poplar (*Populus cathayana* Rehder)	Wu et al., 2015
*Funneliformis mosseae*, *Rhizophagus intraradices* and *Claroideoglomus etunicatum*	Desert grass (*Panicum turgidum* Forssk.)	Hashem et al., 2015
*Rhizophagus intraradices, Claroideoglomus etunicatum* and *Septoglomus constrictum*	Maize (*Zea mays* L.)	Estrada et al., 2013
*Funneliformis mossseae*	*Suaeda salsa* L.	Li et al., 2012
*Funneliformis mossseae*	Wheat (*Triticum aestivum* L.)	Abdel-Fattah and Asrar, 2011
*Rhizophagus intraradices*	*Daucus carota* L.	Hammer and Rillig, 2011
*Funneliformis mosseae*	Maize (*Zea mays* L.)	Sheng et al., 2011
*Funneliformis macrocarpum*	*Sesbania aegyptiaca*, *Sesbania grandiflora*	Giri and Mukerji, 2004
*Claroideoglomus claroideum*	Olive (*Oleae uropaea*)	Porras-Soriano et al., 2009
**Rhizobia**		
*Bradyrhizobium* sp.	Stylo (*Stylosanthes guianensis*)	Dong et al., 2017
*Bradyrhizobium japonicum*	Soybean (*Glycine max*)	Egamberdieva et al., 2017
*Rhizobium* sp.	Rape seed (*Brassica napus*)	Saghafi et al., 2019
*Mezorhizobium ciceri*	Chick pea (*Cicer arietinum*)	Pandey et al., 2018
*Mesorhizobium* sp.	Chick pea (*Cicer arietinum*)	Deepika and Satyavir, 2015
*Rhizobium* sp.	Mung bean (*Vigna radiata*)	Zahir et al., 2010
*Rhizobium* sp.	Lentil (*Lens culinaris*)	Sepúlveda-Caamaño et al., 2018
*Bradyrhizobium* RA-5	Pigeon pea (*Cajanus cajan*)	Bano et al., 2015
*Rhizobium tropici* CIAT 899	Maize (*Zea mays* L.)	Fukami et al., 2018
*Rhizobium* sp.	Maize (*Zea mays* L.)	Bano and Fatima, 2009
*Rhizobium tropici* CIAT899	Common bean (*Phaseolus vulgaris*)	Dardanelli et al., 2008
**Mycorrhizal fungi & Rhizobia**		
*Funneliformis mosseae* and *Agrobacterium pusense* YIC4105	*Sesbania cannabina*	Ren et al., 2016
*Rhizophagus intraradices* C *Bradyrhizobium*sp. Aust11c	*Acacia auriculiformis*	Diouf et al., 2006

### Arbuscular Mycorrhizae in Salt Stress Amelioration

AM fungi can establish a symbiotic relationship with approximately 80% of terrestrial plant species, including crops (Berruti et al., 2016). These microsymbionts exist by obtaining nutrients from plants, and can effectively help plants to obtain water and nutrients needed for growth from the soil. In addition, they can improve the ability of plants to resist abiotic stress (Shabala and Pottosin, 2014). In brief, AM fungi increase the osmotic potential of root cells by enhancing the host plant’s water absorption, nutrient intake, and accumulation of osmotic adjustment substances, thereby reducing salt stress on the host plant. Studies have shown that the formation of arbuscular mycorrhizae can reduce the absorption of Cl^–^ ions by root tissues and at the same time prevent the transfer of Na^+^ to upper stem and leaf tissues under high salinity (Evelin et al., 2009). Under natural conditions, AM fungi can survive in high-concentration saltwater environments (Evelin et al., 2009). For instance, AM fungi were found in the heavily saline-alkali soil of the Tabriz Plain, Iran, with a soil salinity up to 92.0 dS/m (Aliasgharzad et al., 2001). The effect of AM fungi on plant salt tolerance has been studied in many plants, including giant reed (Romero-Munar et al., 2019), *Sesbania* (Kong et al., 2017; Ren et al., 2018), *Zea mays* (Sheng et al., 2011; Krishnamoorthy et al., 2016), cucumber (Hashem et al., 2018), olive (Porras-Soriano et al., 2009), *Chrysanthemum morifolium* (Wang et al., 2018), durum wheat (Fileccia et al., 2017), rice (Porcel et al., 2016), desert grass (Hashem et al., 2015) and tomato (Khalloufi et al., 2017). In mycorrhizal plants, AM fungi improved salt tolerance, helped maintain normal growth and yield under salt stress (Elhindi et al., 2017; Fileccia et al., 2017; Wang et al., 2018), nutrient absorption capacity (Krishnamoorthy et al., 2016; Elhindi et al., 2017), photosynthesis capability (Hashem et al., 2015; Shamshiri and Fattahi, 2016; Chen et al., 2017), and proline content, and promoted the accumulation of soluble sugars in roots. Under salt stress, the colonization of arbuscular mycorrhizae significantly increased the biomass of *Sesbania* (Kong et al., 2017); at 100 mM salinity, the biomass increased by 431%. It has also been reported that AM fungus inoculation has a similar growth-promoting effect on sweet sorghum, and can promote better biomass production than in plants without AM fungus inoculation in a salt environment (Wang et al., 2019). These benefits of AM fungi under saline conditions depend on the symbiotic associations formed by specific strains and plants (Table 1); therefore, it is necessary to select efficient fungal strains for certain plants.

### Rhizobia Help Legumes Adapt to Saline Conditions

*Rhizobium* is a genus of Gram-negative multi-source soil bacteria that can form nodules on the roots of legumes. These bacteria exist in special root nodule cells and provide nitrogen for plant growth by fixing N_2_ from the atmosphere, while at the same time the plant provides a carbon source for their growth (Pawlowski and Demchenko, 2012). Many studies have shown that inoculating suitable rhizobia strains can increase the dry weight of legumes under salt stress conditions, including *Sesbania cannabina* (Ren et al., 2016), *Stylosanthes guianensis* (Dong et al., 2017), chickpea (Deepika and Satyavir, 2015), pigeon pea (Bano et al., 2015), and soybean (Egamberdieva et al., 2017). This growth-promoting effect comes from an effective symbiotic relationship. Ethane reduction activity can be detected even under high salt conditions, but depends on the specific rhizobia–legume symbiosis combination (Bala et al., 1990; Elsheikh and Wood, 1995). Studies have shown that under salt stress conditions, salt-tolerant rhizobia strains can form a functional symbiosis with *S. cannabina*, and soybean (Ren et al., 2016; Egamberdieva et al., 2017), while salt-sensitive strains cannot. These results indicate that the inoculation of salt-tolerant rhizobia can improve biological nitrogen fixation under salt stress conditions. Numerous studies have shown that fast-growing rhizobia are more salt-tolerant than slow-growing rhizobia. Strains of the genus *Rhizobium* are generally more salt-tolerant than those of the genus *Bradyrhizobium*. Therefore, inoculating symbiotic strains with stronger salt tolerance under salt stress conditions may better promote the growth and yield of host plants (Zahran, 2001).

Several salt-tolerant rhizobia have been isolated that can tolerate high salt environments (Oshone et al., 2013; Srivastava et al., 2013). Some of these strains can grow at NaCl concentrations exceeding 350 mM (Dong et al., 2017). The salt tolerance of rhizobia is related to the accumulation of various osmotic adjustment substances in their cells (Del Cerro et al., 2019). These osmotic regulators include K^+^, glutamic acid, proline, glycine betaine, proline betaine, trehalose, dipeptide N-acetyl glutamine, and poly β-hydroxybutyrate. Their protective effects on rhizobia cells under high-salt conditions have been reported one after another. Studies have found that in *R. meliloti* salt-tolerant strains, the glycine betaine content is higher than insensitive strains (Dong et al., 2017). It is also believed that IAA synthesis by rhizobia can prevent the harmful effects of salinity. Bianco and Defez (2009) reported an IAA over-yielding mutant of *Sinorhizobium meliloti*, which significantly increased the tolerance of *Medicago truncatula* to salt stress. Compared with the wild-type strain, the proline content and accumulation of antioxidant enzymes were higher in plants inoculated with the mutants. Inoculation with these symbiotic bacteria can help the host plants effectively resist salt stress. For example, it has been reported that inoculation of *B. japonicum* S2492 significantly increased the dry weight, plant height, and yield (> 35%) of soybeans in arid saline soil (Egamberdiyeva et al., 2004).

**FIGURE 2:**
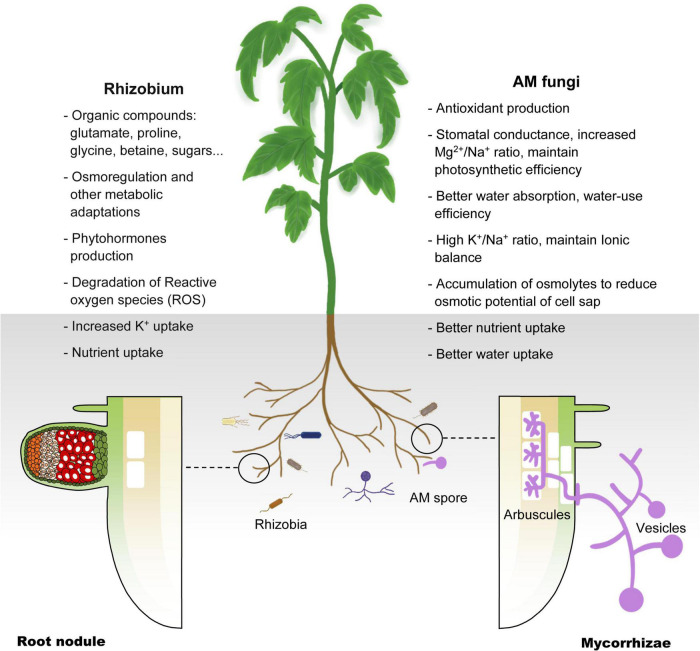
Schematic illustration of the mechanisms deployed by AM fungi and Rhizobium in host plant coping with salinity stress. Salinity impedes plant absorption of water and nutrients, resulting in physiological drought. AM and Rhizobium help plants in salt stress by improving osmoregulation, antioxidant production, K^+^ uptake and other nutrient uptake (see text).

In summary, previous studies have shown that selected AM fungi and rhizobia strains that are compatible with plants can be used to improve the salt tolerance of crops and plants used for saline soil remediation. However, for crops used in saline agriculture, a lack of compatibility with these microsymbionts is likely to become the bottleneck of this new agricultural technology. To fully exploit the beneficial effects of plant–microsymbiont associations, we need to understand the molecular mechanism of symbiosis between plants and these symbionts (Figure 2).

## Depth Horizon: Understanding the Symbiotic Molecular Network in Plants

Symbiotic microorganisms that promote plant abiotic (salt) stress resistance occur naturally and exist widely, such as AM fungi and *Rhizobium*. However, traditional crop breeding techniques, including genetic engineering, domestication and crossbreeding, do not consider the role of symbiotic microorganisms in promoting stress resistance. Moreover, the application of new genetic engineering techniques in breeding overlooks the perspective of plant–microbial symbiosis. Fortunately, research on plant–microbial symbiosis has been continuous and fruitful. There has been considerable research progress on key genes that regulate the process of symbiosis establishment between plants and microorganisms. Among them, the most exciting one is a common symbiotic pathway may be exist in plants (Oldroyd, 2013). Kistner et al. (2005) found that a single gene mutation can inhibit both bacterial and fungal infection of plant root tissue. At the same time, numerous studies of Ca^2+^ signaling in nuclei have shown that it can mediate calcium oscillations of varying intensity (Yuan et al., 2017; Poovaiah and Du, 2018; Plasencia et al., 2021), to activate different downstream pathways. This may be the reason why CCaMK protein, core component in the common symbiotic pathway, can be activated differentially, e.g., mycorrhizal or nodular formation (Russo et al., 2013). These findings could become a key starting point for the use of new gene-editing technologies to engineer non-legumes to establish better symbiotic relationships with versatile symbiotic microorganisms.

### The Common Symbiosis Pathway (SYM) in a Mutually Beneficial Symbiosis With AM Fungi or *Rhizobium*

**FIGURE 3:**
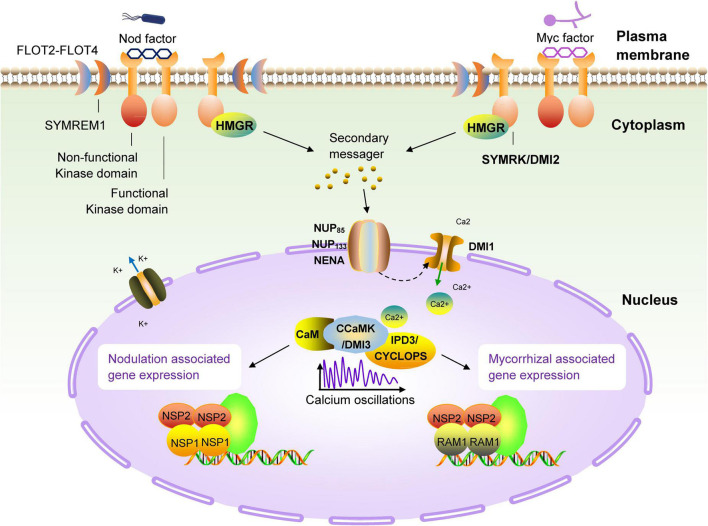
Schematic diagram of the common symbiotic pathway (SYM) of nodulation and arbuscular mycorrhizal signals in legumes. The first known molecular component in the symbiotic pathway (SYM) signaling pathway is the receptor-like kinase DMI2 of *M. truncatula* (homologous to SYMRK of *L. japonicus*) (Kevei et al., 2007; Antolín-Llovera et al., 2014). The direct target of DMI2 is unclear, but it can be determined that the signal is transmitted to the nuclear membrane by a secondary messenger. There are three nucleoporins (NUP85, NUP133 and NENA) in *L. japonicus*, which continue to transmit symbiotic signals to the nucleus (Genre and Russo, 2016). There is a recognized cation channel DMI1 on the nuclear membrane, the results have confirmed the role of DMI1 in the formation of Ca^2+^ oscillations, which has also been confirmed to participate in the conduction of this signal into the nucleus (Charpentier et al., 2008). In the nucleus, the calmodulin-dependent kinase DMI3 (CCaMK in *L. japonicus*) is responsible for decoding the induced calcium oscillation signal (Gleason et al., 2006; Tirichine et al., 2006), and then, together with IPD3 (homolog of CYCLOPS in *L. japonicus*) (Yano et al., 2008; Singh et al., 2014), it takes over the upstream signal and activates a set of downstream transcriptional regulators (NSP1, NSP2, and RAM1) (Kaló et al., 2005; Smit et al., 2005; Hirsch et al., 2009; Pimprikar et al., 2016), these regulatory factors regulates the expression of genes related to nodulation and arbuscular mycorrhiza in the terminal, respectively.

Because legumes can establish symbiotic relationships with AM fungi and rhizobia at the same time, people have carried out extensive research on legumes from the perspective of symbiotic molecular mechanisms. In the past few decades, genetic studies on legumes and AM fungi have successively identified the genetic components necessary for the establishment of a symbiotic relationship. These genes constitute the molecular basis of the relationship between most terrestrial plants, including gramineous plants, and microsymbionts, and are now collectively referred to as the common symbiotic pathway (MacLean et al., 2017; de Bruijn, 2020; Figure 3). Studies have found that AM fungi use the so-called ‘Myc factor’ to stimulate plant roots to begin a dialogue and eventually form a symbiotic relationship (Rasmussen et al., 2016; Pimprikar and Gutjahr, 2018). The chemical composition of the Myc factor is lipochitooligosaccharides (LCOs), secreted by AM fungi and released into the rhizosphere of plants. The Myc factor is sensed by the LysM receptor kinase present on the plant root cell membrane (Gough et al., 2018; He et al., 2019). The transduction of Myc factor signals into plant cells triggers the AM symbiosis signaling pathway. The currently identified components of this pathway are an LRR receptor kinase (MtDMI2/LjSYMRK), a nuclear cation channel protein (MtDMI1/LjPOLLUX, LjCASTOR), a nucleoporin protein (LjNUP85, LjNUP133, NENA), a calcium pump protein (MtMCA8), a calcium-dependent and calmodulin-dependent protein kinase (MtDMI3/LjCCaMK) and its interacting protein components (MtIPD3/LjCYCLOPS) and two GRAS-family transcription factors, NSP2 and RAM1 (Venkateshwaran et al., 2012; Binder and Parniske, 2013; Xue et al., 2015; Gough et al., 2018; Grosche et al., 2018; Hakoshima, 2018; Li et al., 2018; Pimprikar and Gutjahr, 2018). Studies have proved that the above components are all necessary for the establishment of symbiosis. Mutant plants containing non-functional genes cannot form a sound symbiotic structure. Many related studies have found that most terrestrial plants (including non-legumes such as corn and rice), and even some lower plants (such as mosses), contain AM symbiotic signaling pathway-related genes. This phenomenon shows that plants have evolved for symbiosis and that the emergence of the ‘molecular machinery’ related to symbiosis in plants has a very ancient origin (Rimington et al., 2018; Delaux and Schornack, 2021).

Leguminous plants are the second most diverse group among terrestrial plants. In addition to symbiosis with AM fungi, they can also establish effective symbiotic relationships with *Rhizobium* species. This relationship appears in the form of root nodules. In rare cases, the symbiosis can form stem nodules, such as in *Azorhizobium caulinodans* and *Sesbania* (Lee et al., 2008; Liu et al., 2017). Nodules are a group of highly specialized plant cells wrapped under the epidermis of plant roots. Their purpose is to provide shelter for the bacteria. Inside, the rhizobia efficiently fix nitrogen from the atmosphere and exchange nutrients with the plant, forming a close relationship. Related genetic studies on legumes have shown that the ancient SYM pathway has further specialized in legumes. The nodulation ability of some legume mutants with defective AM symbiosis as mentioned above is also affected, which indicates the high degree of homology of the SYM pathway in the establishment of different forms of symbiosis between plants and microorganisms (Kistner et al., 2005). In addition to the known members of the SYM pathway, other molecular components related to the establishment of symbiosis have high homology. For example, NSP1 is an important component of rhizobia–legume symbiosis, its original copy (RAM1) is also critical in AM symbiosis, and it seems to belong to a broader SYM pathway (Gobbato et al., 2012; Fonouni-Farde et al., 2016; Floss et al., 2017; Lace and Ott, 2018). Detailed studies have discovered more and more key molecular components involved in the symbiosis of rhizobia and plants, and gradually revealed an ancient truth: these newly discovered components are part of or modified from the SYM pathway. The symbiotic interaction between legumes and rhizobia begins with the host’s perception of microbial signal molecules called ‘nodulation factors’ (NFs) released by rhizobia (Buhian and Bensmihen, 2018). Notably, Nod factors are also LCOs, which are recognized and bound by LysM-type receptor kinases in legumes (Buendia et al., 2018; Gough et al., 2018; Bozsoki et al., 2020). Studies have shown that NFs directly binds to the NF receptors NFR5 and NFR1, which are located on the cell membrane of *Lotus japonicus*. The dissociation constant (Kd) value of NF-ligand binding is in the nanomolar range (about 10 nM for NFR5 and about 5 nM for NFR1), which is close to the concentration known to trigger the onset of symbiosis (Broghammer et al., 2012). The signal cascade required for nodulation is eventually triggered by the perception of LCOs by these plasma membrane receptors and triggers a series of rapid reactions in the host cell, including the formation of ion currents, alkalization of the cytoplasm, production of reactive oxygen species, and nuclear and perinuclear calcium oscillations (Cardenas et al., 2008; Chabaud et al., 2011; Naffah de Souza et al., 2017).

The abovementioned research progress shows that symbiotic genes related to the SYM pathway existed in the common ancestor of land plants, and their functions have remained fundamentally unchanged during the evolution of land plants (Wang et al., 2010; Radhakrishnan et al., 2019). Moreover, increasing numbers of related studies have revealed that this common symbiotic pathway (SYM) is highly conserved in different symbiotic relationships, and even the interactions of plants with microbes other than mycorrhiza and nodules are associated with components of the SYM pathway (Chiu and Paszkowski, 2020). The discovery of the SYM pathway and continuing research progress provide a promising strategy for genetically engineering symbiotic molecular components into plants, to utilize symbiotic microorganisms to improve the vigor and salt resistance of host plants.

### Multi-Omics Approaches for Unraveling the SYM Pathway and Identification of Anchor Genes

In the past two decades, research on clarifying the molecular mechanism of plant–microbe symbiosis has not only successfully identified individual key genes involved in the establishment of symbiosis through reverse genetics but also made much progress through high-throughput genomics and proteomics quantitative strategies (Genre and Russo, 2016; Lardi and Pessi, 2018; Rehman et al., 2019; Chiu and Paszkowski, 2020; diCenzo et al., 2020; Tong et al., 2020). With the completion of genome sequencing for a large number of plants (including legumes and non-leguminous crops), it has become more feasible to apply the “-omics” strategy to the study of symbiosis (Lardi and Pessi, 2018). Regarding the use of proteomics and phosphorylation proteomics to study the symbiosis signal of legumes, Rose et al. (2012) used a proteomics method based on deep non-targeted mass spectrometry and found that the phosphorylation status of 13,506 phosphorylation sites of 7,739 *M. truncatula* proteins changed rapidly under NF treatment. The study also found that in the early stage of symbiotic signal transmission (within 1 h after NF induction), the types and contents of proteins did not change. The rapid cellular and molecular response induced by NF mainly relied on post-translational modifications, such as phosphorylation, rather than protein synthesis or degradation (Rose et al., 2012). This study also employed a genetics method to discover some new symbiosis-related candidate genes using a limited number of key gene loss-of-function mutants in the SYM pathway, which may be involved in symbiosis signaling, cell cycle regulation, and root hair growth and infection (Rose et al., 2012). Similarly, Nguyen et al. (2012) analyzed the phosphoproteome during the rhizobial colonization of soybean root hair cells to understand the molecular mechanism of nodule formation. Their study identified 1,126 phosphorylated soybean proteins and detected 1,659 phosphorylation sites, and in soybean plants inoculated with *Bradyrhizobium japonicum*, 273 phosphorylation sites of 240 phosphorylated proteins were significantly altered, many of which were the same phosphorylation sites identified by Rose et al. (2012). In addition, a genome-wide transcriptome study aimed at elucidating the Myc factor signal transduction process in *M. truncatula* found that all kinds of symbiotic LCO analogs upregulated a common set of genes (Czaja et al., 2012). On an even larger scale, the three plant systems *Casuarina glauca*, *M. truncatula* and *Oryza sativa* were used to compare the transcriptomes of genes involved in rhizobial nodulation, actinomycete nodulation, and arbuscular mycorrhiza formation. The results showed that a group of common genes was regulated in these three endosymbiosis processes (Tromas et al., 2012). These systemic approaches together provide a rapid means of filling the gaps in our knowledge on symbiotic signal transduction as well as potentially pointing us in new directions with genes and proteins not previously thought to be involved. “Multi-omics approaches” together enable the rapid description of the mechanism of symbiotic signal transduction, potentially providing a blueprint for genetically engineering crops to utilize the potential of symbiotic microorganisms to promote growth and resist stress.

## Possibility of Pairing Non-Legume Crops and Symbiotic Microbes to Cope With Salt Stress Through Gene Editing

**FIGURE 4:**
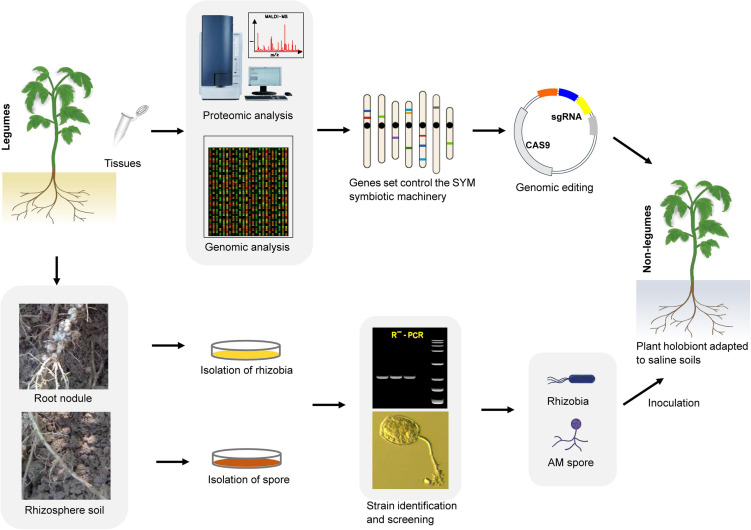
An integrated tool for developing plant ‘holobiont’ adapted to saline. First, through multi-omics approaches to identify ‘anchor genes’ in the SYM pathway. Then, using genome editing tool such as CRISPR/Cas9 to engineer non-leguminous crops to associate better with rhizobia and AM fungi utilize the SYM symbiotic machinery. In parallel, screening of highly efficient salt-tolerant improving strains from the rhizosphere of saline-alkali soil plants. At last, in combing genetically modified crops that are easy to establish symbiotic relationships with specific AM fungi and rhizobia strains to develop a plant ‘holobiont’, which can better adapt to the salt soil environment.

Global human activities and climate change are intensifying, leading to further intensification of cultivated land salinization. To expand the planting area on saline soils, increase the net yield of crops, and avoid the impact of negative environments on crop yields while reducing the dependence of large-scale shallow-root monocropping crops on chemical fertilizers, a promising strategy is to make full use of growth- and salt resistance-promoting symbiotic microorganisms such as AM fungi and rhizobia. To achieve this goal, we need to fully understand the molecular mechanism of symbiosis between plants (especially legumes) and microorganisms. One specific method is to use the symbiosis mechanism of SYM (the relevant molecular components of this mechanism already exist in most land plants) together with genetic engineering methods to modify the main food crops (legumes and non-legumes) so that they can establish better symbiotic relationships with microsymbionts, and achieve high-quality microsymbiosis through screening to construct a plant ‘holobiont’ (Figure 4). This will allow us to make full use of the growth-promoting and anti-stress functions of microsymbionts to enable the sustainable development of saline agriculture under future climate change conditions. Because most terrestrial plants (including cereals) already contain the relevant molecular components of the SYM symbiosis mechanism, realizing the perception of rhizobial signals is the key first step to initiating the SYM pathway and achieving infection in non-legume plants. Studies on *Parasponia* have shown that successful cell colonization can enable a certain degree of nitrogen fixation without the formation of complex nodule organs (Geurts et al., 2012), which implies that the microsymbiont and the host are also carrying out the exchange of nutrients and physiological signals in an incomplete symbiotic relationship with only infection achieved. Unlike NF signaling, the receptors specifically used for Myc factor sensing have not yet been determined. Although the LysM type receptor kinase LYR1 is strongly upregulated during AM symbiosis in *M. truncatula* (Arrighi et al., 2006; Benedito et al., 2008; Young et al., 2011), there is still a lack of direct experimental evidence to support its role in Myc factor perception. Myc factors can trigger calcium oscillation in both legumes and non-legume dicots, but there is no such response in monocots. However, studies have shown that there is crosstalk between the NF and Myc factor signaling pathways in the same plant (Groth et al., 2010; Gully et al., 2018; Suzaki et al., 2019). This phenomenon indicates that it is possible to modify the signal receptors in plants to make them sense the NF and Myc factors at the same time. In the natural environment, only a few monocotyledonous and dicotyledonous plants can form close and effective associations with rhizobium. However, most of the research aimed at using symbiotic microorganisms to promote stress resistance in plants has focused on screening out more effective microbial strains. The specific molecular mechanism encoded in the plant genome tightly controls the interaction with these beneficial microsymbionts. We have shown in this review that plants continue to use this ancient and extensive symbiotic molecular machinery (the SYM pathway) in their evolution to recruit AM fungi and a variety of tolerance-promoting bacteria to adapt to environmental stress. This fact demonstrates that utilizing microsymbiosis by artificially modifying the key components of the SYM pathway in target crops is an achievable goal. For example, the transformation of economically valuable non-legume crops to expand beneficial microsymbiosis in the soil will have a huge impact on the sustainability of food amidst the gradual salinization of arable land. Therefore, the combination of ongoing high-throughput research and in-depth genetic analysis will be of vital importance for engineering salt tolerance-promoting associations in non-leguminous crops and particularly in cereals.

## Concluding Remarks

It is clear that expansion of conventional agricultural practices to meet future demands amidst the gradual salinization of farmland is neither economically nor environmentally feasible. Therefore, saline agriculture urgently needs innovations to follow on from the Green Revolution to sustainably meet the demand for global food security. One way to improve the production efficiency of crops under saline conditions is to expand the growth-promoting and stress-resistance effects of microsymbionts. These microsymbionts include AM fungi and a variety of rhizobia that can improve crop growth and vitality, nutrient utilization efficiency, and biotic/abiotic stress tolerance. However, traditional crop breeding technologies (including genetic engineering technology, domestication, and hybrid breeding) rarely consider the role of symbiotic microorganisms in promoting plant stress resistance, and breeding using new genetic engineering technologies overlooks the perspective of plant–microbe symbiosis. Fortunately, however, research on plant–microbe symbiotic relationships have been continuous and fruitful. In the past few decades, researchers have successfully identified the key molecular components of arbuscular mycorrhiza and nodule formation with plants through genetic studies. These genes are collectively referred to as the SYM pathway. Through multi-omics methods, analysis of these key symbiotic components has continuously deepened. Based on these findings, we may use new gene-editing technologies such as the clustered regularly interspaced short palindromic repeat (CRISPR)/CRISPR-associated protein 9 (Cas9) system to customize plant utilization of the SYM symbiotic machinery (which is present already in most land plants and, in particular, in cereals), in combination with AM fungi and rhizobia strains to develop salt-tolerant plant ‘holobiont’. These developments will open a new avenue for capitalizing on symbiotic microorganisms to strengthen plant salt resistance. Agricultural practices and production efficiency under saline conditions will be greatly improved to meet the increasing global food demand.

## Author Contributions

C-GR analyzed the data and wrote the manuscript. Z-YL, C-CK, Z-HZ, X-LW, J-CY and SQ participated in revisions of the manuscript. All authors have read the manuscript and approved the final version of the manuscript. Special thanks to Wang Yu for his hand drawing in Figure 1.

## Conflict of Interest

The authors declare that the research was conducted in the absence of any commercial or financial relationships that could be construed as a potential conflict of interest.

## Publisher’s Note

All claims expressed in this article are solely those of the authors and do not necessarily represent those of their affiliated organizations, or those of the publisher, the editors and the reviewers. Any product that may be evaluated in this article, or claim that may be made by its manufacturer, is not guaranteed or endorsed by the publisher.
